# Physical activity and nutrition behaviour outcomes of a cluster-randomized controlled trial for adults with metabolic syndrome in Vietnam

**DOI:** 10.1186/s13063-016-1771-9

**Published:** 2017-01-13

**Authors:** Van Dinh Tran, Andy H. Lee, Jonine Jancey, Anthony P. James, Peter Howat, Le Thi Phuong Mai

**Affiliations:** 1 Department of Community Health and Network Coordination, National Institute of Hygiene and Epidemiology, No. 1, Yersin Street, Hanoi, Vietnam; 2 School of Public Health, Curtin University, Perth, WA 6845 Australia; 3 Collaboration for Evidence, Research and Impact in Public Health, Curtin University, Perth, WA 6845 Australia; 4 Curtin Health Innovation Research Institute, Curtin University, Perth, WA 6845 Australia

**Keywords:** dietary behaviours, health promotion, metabolic syndrome, physical activity, randomized controlled trial, Vietnam, walking

## Abstract

**Background:**

Metabolic syndrome is prevalent among Vietnamese adults, especially those aged 50–65 years. This study evaluated the effectiveness of a 6 month community-based lifestyle intervention to increase physical activity levels and improve dietary behaviours for adults with metabolic syndrome in Vietnam.

**Methods:**

Ten communes, involving participants aged 50–65 years with metabolic syndrome, were recruited from Hanam province in northern Vietnam. The communes were randomly allocated to either the intervention (five communes, *n* = 214) or the control group (five communes, *n* = 203). Intervention group participants received a health promotion package, consisting of an information booklet, education sessions, a walking group, and a resistance band. Control group participants received one session of standard advice during the 6 month period. Data were collected at baseline and after the intervention to evaluate programme effectiveness. The International Physical Activity Questionnaire – Short Form and a modified STEPS questionnaire were used to assess physical activity and dietary behaviours, respectively, in both groups. Pedometers were worn by the intervention participants only for 7 consecutive days at baseline and post-intervention testing. To accommodate the repeated measures and the clustering of individuals within communes, multilevel mixed regression models with random effects were fitted to determine the impacts of intervention on changes in outcome variables over time and between groups.

**Results:**

With a retention rate of 80.8%, the final sample comprised 175 intervention and 162 control participants. After controlling for demographic and other confounding factors, the intervention participants showed significant increases in moderate intensity activity (*P* = 0.018), walking (*P* < 0.001) and total physical activity (*P* = 0.001), as well as a decrease in mean sitting time (*P* < 0.001), relative to their control counterparts. Significant improvements in dietary behaviours were also observed, particularly reductions in intake of animal internal organs (*P* = 0.001) and in using cooking oil for daily meal preparation (*P* = 0.001).

**Conclusions:**

The prescribed community-based physical activity and nutrition intervention programme successfully improved physical activity and dietary behaviours for adults with metabolic syndrome in Vietnam.

**Trial registration:**

Australian New Zealand Clinical Trials Registry, ACTRN12614000811606. Registered on 31 July 2014

**Electronic supplementary material:**

The online version of this article (doi:10.1186/s13063-016-1771-9) contains supplementary material, which is available to authorized users.

## Background

Metabolic syndrome is a cluster of risk factors for cardiovascular disease and type 2 diabetes that includes abdominal obesity, elevated blood pressure, reduced high-density lipoprotein cholesterol levels, elevated fasting triglyceride and high glucose concentrations [1]. Metabolic syndrome is becoming a global epidemic [2] and is often undiagnosed [3, 4], with about one-quarter of the adult population worldwide affected by the condition [5]. In Vietnam, it has been reported that almost two-fifths of adults aged 35–65 years have metabolic syndrome [6]. A recent cross-sectional study found that 16.3% of the Vietnamese population aged 40–64 years have metabolic syndrome, while those aged 55–64 sustain the highest prevalence and account for 27% of the cases diagnosed [7].

Modifiable lifestyle factors, such as physical inactivity and unhealthy dietary habits, are associated with the development of metabolic syndrome [6–8]. It is estimated that 28.7% of Vietnamese adults are insufficiently active (<600 metabolic equivalent tasks (MET), min per week) [9]. Moreover, the household food consumption pattern has changed rapidly [10], with increases in intake of dietary sodium and saturated fat [10, 11]. The proportion of energy intake from fat has doubled from 8.4% to 17.6% in the last two decades [10]. It has been reported that physical inactivity and insufficient vegetable and fruit consumption are responsible for 0.7% and 3.07%, respectively, of the total burden of disease in Vietnam. These unhealthy lifestyle behaviours have also contributed to over 5% of deaths from non-communicable disease [9]. In recognition of the high mortality and morbidity associated with non-communicable disease in Vietnam, the National Strategy for Non-Communicable disease Control and Prevention 2015–2025 was established to reduce behavioural risk factors, such as smoking, alcohol consumption, physical inactivity and salt consumption [12].

Interventions that use a combination of physical activity training and dietary modification have been recommended for metabolic syndrome [3, 13]. A recent meta-analysis concluded that interventions that motivate participants to improve lifestyle behaviours and weight management are essential for controlling metabolic syndrome risk factors [14]. Reported outcomes of intervention strategies designed to improve physical activity and dietary behaviours vary in terms of effectiveness [14]. However, a systematic review found that participation in walking groups provides an effective way of increasing physical activity and is suitable for any age group, especially older adults [15]. Walking, as a moderate activity, is the most popular leisure activity across all socio-economic groups [16, 17]. Walk leaders, who are either volunteers or nominated by their group members, have been demonstrated to play a key role in motivating participants to become physically active [16, 18].

With regard to resources, interventions that incorporate an information booklet to improve knowledge are found to be effective [19–21]. For example, a recent study in rural Western Australia that made use of an information booklet achieved positive changes in physical activity and dietary behaviours for participants with or at risk of metabolic syndrome [22]. Furthermore, personal feedback and group support are important for lifestyle interventions to control metabolic syndrome and its risk factors [14].

In view of the high prevalence of metabolic syndrome among middle-aged people in Vietnam [7, 9, 23], the Vietnam Physical Activity and Nutrition programme was designed to target adults aged 50–65 years with metabolic syndrome. The aim of this study was to determine whether implementation of the Vietnam Physical Activity and Nutrition programme was effective in terms of improving physical activity levels and dietary behaviours of its participants after a 6 month intervention.

## Methods

### Study design

**Fig. 1:**
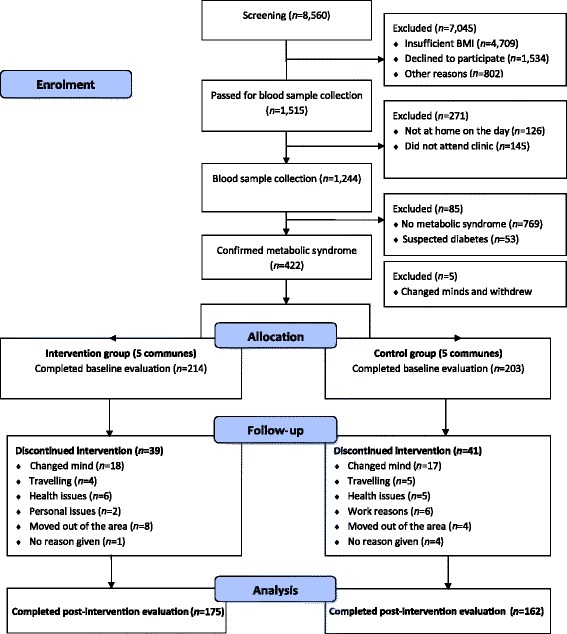
CONSORT flow chart. BMI, body mass index

The protocol of the Vietnam Physical Activity and Nutrition programme has been described in detail previously [24], in accordance with the Consolidated Standards of Reporting Trials (CONSORT) Statement (see Fig. 1 for the CONSORT flow chart and Additional file 1 for the CONSORT checklist of the trial). It was a 6 month community-based cluster-randomized controlled trial targeting adults aged 50–65 years with metabolic syndrome from 10 communes in Hanam province, northern Vietnam. Outcomes were collected from intervention and control groups at baseline and post-intervention testing. The trial was registered with the Australia and New Zealand Clinical Trial Registry (ACTRN12614000811606). The research protocol was approved by the Curtin University Human Research Ethics Committee (approval number: HR139/2014). Written informed consent was sought from each participant prior to entry in the trial.

### Participants

Adults aged 50–65 years with metabolic syndrome were recruited and invited to participate in the study. Metabolic syndrome status was determined based on the modified National Cholesterol Education Programme Adult Treatment Panel III criteria of having three of the five risk factors [25]: (1) large waist circumference (male ≥90 cm, female ≥80 cm, for Asian population [1]; (2) raised triglyceride levels (≥1.7 mmol/l or 150 mg/dl); 3) reduced high-density lipoprotein cholesterol (male <1.03 mmol/l or 40 mg/dl, female <1.29 mmol/l or 50 mg/dl); (4) raised blood pressure (systolic ≥130 mmHg or diastolic ≥85 mmHg); and (5) raised fasting plasma glucose level (≥6.1 mmol/l or ≥110 mg/dl).

Exclusion criteria were suspected type 2 diabetes (fasting plasma glucose level ≥7.1 mmol/l); treatment or a history of treatment for type 2 diabetes, cardiovascular disease, dyslipidaemia, hyperglycaemia, and hypertension; or involvement in a physical activity or dietary programme within the previous year.

### Procedure

The participant selection phase, including initial screening and determination of metabolic syndrome status, occurred between October 2014 and January 2015, and the post-intervention evaluation was completed in November 2015.

#### Screening

A total of 8560 adults aged 50–65 years residing in 10 randomly selected communes within Hanam province were contacted, and invited to attend their local commune health centre for screening. Small incentives (reimbursement of transport expenses) were provided to encourage attendance. At these sessions, a short interview was conducted to obtain information about each participant’s age, sex, physical activity levels, and medication history. The participant’s height and weight were also measured. Body mass index was calculated and classified according to the World Health Organization (WHO) criteria for Asian populations, with body mass index ≥23 being classed as ‘overweight’ [26]. Eligible people with body mass index ≥23 were invited to participate in the next stage of screening.

#### Determining metabolic syndrome status

As shown in Fig. 1, 1515 eligible subjects were invited for blood testing and measurement of waist circumference and blood pressure to confirm their metabolic syndrome status. A formal letter of invitation was delivered to eligible participants. The letter provided detailed information about the time, location, and guidelines for fasting overnight (except for water after 9 p.m. and on the morning of blood sample collection). However, only 1244 people attended the clinic for blood sample collection and anthropometric measurements. Among them, 422 met the metabolic syndrome criteria and were invited for baseline evaluation. Five individuals changed their minds and subsequently withdrew, leaving a total of 417 participants who completed the baseline assessment.

#### Allocation to control and intervention groups

The 10 selected communes were randomly allocated to either the intervention group (five communes, *n* = 214) or the control group (five communes, *n* = 203) by a member of staff at Hanam Provincial Preventive Medicine Centre using a table of random numbers. The intervention group underwent the Vietnam Physical Activity and Nutrition programme, whereas the control group participants, who were fully aware of their status, received one session of standard advice and were wait-listed to receive the intervention package following completion of the post-intervention test. At the end of the 6 months period, 175 intervention (response rate 81.8%), and 162 control participants (response rate 79.8%) completed the post-intervention test assessment; see Fig. 1.

### Intervention

The intervention was developed and underpinned by social cognitive theory [27, 28]. It was designed to promote physically activity and the maintenance of a healthy diet to participants. The Vietnam Physical Activity and Nutrition programme included four education sessions, a booklet, a resistance band and walking groups. All components of the Vietnam Physical Activity and Nutrition programme were conducted within the participants’ communes to minimize subject burden. Participants attended four 2-hour education sessions at months 1, 2, 3 and 4 of the intervention, and participated in walking groups established at each commune for 6 months. During the first education session, each participant was provided with the health promotion booklet and a resistance band for strength exercises. Programme staff at the Hanam Provincial Preventive Medicine Centre, trained by the first author, conducted the education sessions, led the walking groups and collected data from participants at baseline and post-intervention testing. These trained walk leaders were provided with a package containing the education materials, as well as a manual for managing the group walks. The walk leaders mobilized participants for walking and encouraged them to achieve physical activity and diet goals. Details of the intervention materials are described elsewhere [24].

### Variables

Demographic and personal information such as age, sex, occupation, marital status, smoking and alcohol consumption was obtained through a structured questionnaire administered to participants via face-to-face interview at baseline testing.

#### Physical activity

The International Physical Activity Questionnaire – Short Form, validated for Vietnamese adults [29], was used to measure physical activity levels, which included vigorous intensity activity, moderate intensity activity, walking and sitting time. In addition, a pedometer (Yamax SW-200, Japan) was given to each intervention participant to count daily steps taken. The device was fitted to the hip and worn for 7 consecutive days at both baseline and post-intervention testing. This objective measure of physical activity has been reported to be accurate and reliable [30].

#### Diet

The brief dietary habits questionnaire was modified from the STEPS questionnaire developed by the WHO [31] to gather information on the consumption of vegetables and fruits, and intake of animal internal organs, as well as the frequency of use of cooking oil and salt for preparing meals.

### Statistical analysis

Descriptive statistics were first applied to summarize the baseline characteristics of the participants by group status. Comparisons between intervention and control participants were undertaken across the two time points using independent samples and paired *t* tests for continuous outcome variables, and the chi-squared test for dichotomous outcomes. For variables with skewed distributions, the Mann-Whitney *U* test and the Wilcoxon signed rank test were applied instead. To accommodate the correlation of observations due to the repeated measures (pre- and post-intervention testing) and the clustering of individuals within the 10 randomly selected communes, multilevel generalized linear mixed models with random effects (participants and communes) were fitted to determine the impacts of intervention on changes in outcome variables over time and between groups [32, 33], while accounting for the effects of potential confounding factors (age, sex, education level, relationship status, occupation, smoking status and alcohol consumption). All statistical analyses were performed in the SPSS package version 21.

#### Binary outcomes

In the presence of many zeros, vigorous activity and moderate activity were dichotomized by participation status (yes, no). For dietary behaviour outcomes, consumption of fruit and vegetables, using cooking oil and salt to prepare meals at least once per day, as well as consumption of animal internal organs more than twice per month, were classified as frequent intake or usage (yes, no). These binary outcomes (vigorous activity, moderate activity, frequent fruit intake, frequent vegetable intake, frequent intake of animal internal organs, frequent use of cooking oil, frequent use of salt) were modelled using logistic mixed regressions.

#### Continuous outcomes

Walking time was considered a continuous variable in metabolic equivalent tasks (MET, min/week). Total physical activity for each individual was calculated by summing across the three activity domains, in which the reported time spent (min/week) was multiplied by the corresponding MET score (8 for vigorous, 4 for moderate and 3.3 for walking) [34]. Sitting time was analyzed in terms of duration (min/week). Generalized linear mixed regression analysis was applied to walking time and total physical activity (MET, min/week), which were logarithmic transformed owing to their positively skewed distributions. A gamma mixed regression model was adopted to analyze the highly skewed sitting time.

## Results

Table 1 presents the characteristics of participants at baseline, with no significant differences observed between the intervention and control groups (*P* > 0.05). The mean age of the participants was 57 (standard deviation, 5) years, with the majority being women. More than 90% of the cohort completed secondary school or higher, and over 90% lived with a partner. Almost one-third of the sample were retired. On average, the participants were slightly overweight, with a mean body mass index of 25.1 (standard deviation, 2).

**Table 1:** Baseline characteristics of intervention and control participants (*n* = 337)

Variable	Intervention group (*n* = 175)	Control group (*n* = 162)	*P* ^a^
Age: mean (standard deviation), years	57.57 (4.93)	57.23 (4.87)	0.52
Weight: mean (standard deviation), kg	60.18 (7.70)	60.32 (7.82)	0.88
Body mass index: mean (standard deviation)	24.97 (1.92)	25.21 (2.29)	0.36
Waist circumference: mean (standard deviation), cm	87.12 (5.62)	87.59 (6.22)	0.47
Hip circumference: mean (standard deviation), cm	94.27 (4.77)	93.70 (6.04)	0.33
Sex			0.36
Female	144 (82.3%)	127 (78.4%)	
Male	31 (17.7%)	35 (21.6%)	
Education level			0.29
Primary school or below	14 (8.0%)	17 (10.5%)	
Secondary school	89 (50.9%)	94 (58.0%)	
High school	45 (25.7%)	34 (21.0%)	
College or university	27 (15.4%)	17 (10.5%)	
Relationship status			0.99
No partner	15 (8.6%)	15 (9.3%)	
With partner	160 (91.4%)	147 (90.7%)	
Occupation			0.55
Farmer or manual worker	41 (23.4%)	49 (30.2%)	
Office job	11 (6.3%)	8 (4.9%)	
Retired	55 (31.4%)	50 (30.9%)	
Business	18 (10.3%)	11 (6.8%)	
Home duties and others	50 (28.6%)	44 (27.2%)	
Smoking status			0.58
Never	154 (88.0%)	138 (85.2%)	
Former	10 (5.7%)	14 (8.6%)	
Current smoker	11 (6.3%)	10 (6.2%)	
Alcohol drinking			0.30
No	145 (82.9%)	127 (78.4%)	
Yes	30 (17.1%)	35 (21.6%)	

^a^Chi-square or *t* test between intervention and control groups

### Physical activity outcomes

Table 2 compares the physical activity outcomes over time and between intervention and control groups. Both groups were similar in terms of physical activity levels at baseline. However, significant improvements (*P* < 0.001) were observed in the intervention group from baseline to post-intervention testing for moderate activity participation, walking time and total physical activity, as well as a reduction in sitting time. There was also a significant increase (*P* = 0.011) of over 5000 steps on average on 7 consecutive days between the two time points. For the control group, no significant change occurred from baseline to post-intervention testing, apart from an apparent decrease in mean sitting time.

**Table 2:** Comparison of physical activity outcomes over time and between intervention and control groups (*n* = 337)

Outcome	Intervention group (*n* = 175)		*P* ^a^	Control group (*n* = 162)		*P* ^b^	*P* ^c^	*P* ^d^
	Baseline	Post		Baseline	Post			
Vigorous activity^e^	22 (12.6%)	12 (6.9%)	0.071	15 (9.3%)	6 (3.7%)	0.042	0.331	0.198
Moderate activity^e^	26 (14.9%)	61 (34.9%)	<0.001	24 (14.8%)	30 (18.5%)	0.371	0.991	0.001
Walking time: mean (standard deviation)^f^	366.3 (396.6)	588.3 (491.3)	<0.001	333.6 (394.3)	326.7 (355.0)	0.680	0.160	<0.001
Total physical activity: mean (standard deviation)^f^	478.5 (496.2)	862.7 (692.5)	<0.001	448.4 (447.9)	502.9 (496.6)	0.260	0.470	<0.001
Sitting time: mean (standard deviation) min/week	2,668.7 (764.0)	1,911.5 (769.8)	<0.001	2,733.5 (807.7)	2,371.2 (963.7)	<0.001	0.450	<0.001
Pedometer: mean (standard deviation), steps/week	48,722 (20,974)	53,882 (20,774)	0.011					

^a^Between baseline and post-intervention tests for intervention group

^b^Between baseline and post-intervention tests for control group

^c^Between intervention and control groups at baseline

^d^Between intervention and control groups at post-intervention testing

^e^Participation of at least 10 min

^f^Non-parametric test applied to MET, min/week

Table 3 summarizes the results of mixed regression analyses of physical activity outcomes pre- and post-intervention. After controlling for commune clustering and the effects of confounding factors, significant improvements among the intervention participants relative to their control counterparts were evident in moderate activity participation (*P* = 0.018), mean walking time (*P* < 0.001), total physical activity (*P* = 0.001) and mean sitting time (*P* < 0.001), according to the group × time interaction term of the mixed regression models. However, no significant change in prevalence of vigorous activity participation was found after the intervention (*P* = 0.643).

**Table 3:** Mixed regression analyses of physical activity outcomes before and after intervention (*n* = 337)

Outcome	Group: intervention		Time: post		Group × Time		Random component	
	Coefficient (95% confidence interval)	*P* ^a^	Coefficient (95% confidence interval)	*P* ^a^	Coefficient (95% confidence interval)	*P* ^e^	*σ* ^f^	*σ* ^g^
Vigorous activity^a^	0.414 (−0.817, 1.645)	0.509	−1.023 (−0.020, −0.026)	0.044	0.297 (−0.962, 1.556)	0.643	0.751	0.714
Moderate activity^a^	−0.143 (−1.325, 1.040)	0.813	0.281 (−0.322, 0.883)	0.361	0.99 (0.169, 1.810)	0.018	0.782	0.501
Walking time^b,d^	−0.039 (−0.126, 0.048)	0.376	0.011 (−0.056, −0.078)	0.745	0.168 (0.080, 0.255)	<0.001	0.044	0.083
Total physical activity^b,d^	−0.032 (−0.129, 0.065)	0.518	0.059 (−0.009, 0.127)	0.091	0.154 (0.063, 0.244)	0.001	0.054	0.114
Sitting time^c^	−0.026 (−0.131, 0.079)	0.627	−0.146 (−0.218, −0.075)	<0.001	−0.191 (−0.291, −0.092)	<0.001	0.054	0.100

^a^Logistic mixed regression model

^b^Linear mixed regression model

^c^Gamma mixed regression model

^d^Logarithmic transformed

^e^Adjusted for age, sex, education level, relationship status, occupation, smoking status and alcohol drinking

^f^Commune random effect

^g^Participant random effect

### Dietary outcomes

Table 4 shows that both groups were similar with respect to the reported dietary behaviour outcomes at baseline, but that the intervention participants appeared more likely to consume fruits than the controls. Significant improvements in some of these dietary outcomes from baseline to post-intervention testing were observed for the intervention group, whereas no apparent changes were found in the control group, apart from a decrease in frequent use of salt for preparing meals. At 6 months, significant differences between groups were demonstrated for all dietary behaviours (*P* < 0.05).

**Table 4:** Comparison of dietary behaviour outcomes over time and between intervention and control groups (*n* = 337)

Outcome	Intervention group (*n* = 175)		*P* ^a^	Control group (*n* = 162)		*P* ^b^	*P* ^c^	*P* ^d^
	Baseline *n* (%)	Post *n* (%)		Baseline n (%)	Post n (%)			
Frequent vegetable intake^e^	164 (93.7)	168 (96.0)	0.333	152 (93.8)	143 (88.3)	0.080	0.966	0.008
Frequent fruit intake^e^	72 (41.1)	85 (48.6)	0.162	47 (29.0)	61 (37.7)	0.099	0.020	0.040
Frequent use of cooking oil^e^	64 (36.6)	36 (20.6)	0.001	41 (25.3)	50 (30.9)	0.266	0.026	0.030
Frequent use of salt^e^	171 (97.7)	90 (51.4)	<0.001	158 (97.5)	115 (71.0)	<0.001	0.910	<0.001
Frequent intake of animal internal organs^f^	49 (28.0)	19 (10.9)	<0.001	37 (22.8)	35 (21.6)	0.789	0.278	0.007

^a^Between baseline and post-intervention tests for intervention group

^b^Between baseline and post-intervention tests for control group

^c^Between intervention and control groups at baseline

^d^Between intervention and control groups at post-intervention testing

^e^At least once per day

^f^More than twice per month

Table 5 summarizes the results of logistic mixed regression analyses of dietary behaviours before and after intervention. After controlling for commune clustering and the effects of confounding factors, the group × time interaction term confirmed significant reductions in frequent intake of animal internal organs (*P* = 0.001) as well as frequent use of cooking oil (*P* = 0.001) by the intervention group relative to the control group over the 6 month period.

**Table 5:** Logistic mixed regression analyses of dietary behaviour outcomes before and after intervention (*n* = 337)

Outcome	Group: intervention		Time: post		Group × Time		Random component	
	Coefficient (95% confidence interval)	*P* ^c^	Coefficient (95% confidence interval)	*P* ^c^	Coefficient (95% confidence interval)	*P* ^c^	*σ* ^d^	*σ* ^e^
Frequent vegetable intake^a^	−0.097 (−1.321, 1.127)	0.876	−0.738 (−1.558, 0.081)	0.077	1.229 (−0.055, 2.514)	0.061	0.603	0.333
Frequent fruit intake^a^	0.67 (−0.333, 1.672)	0.190	0.444 (−0.053, 0.942)	0.080	−0.081 (−0.762, 0.600)	0.816	0.678	0.761
Frequent use of cooking oil^a^	0.246 (−0.805, 1.298)	0.646	0.294 (−0.209, 0.797)	0.252	−1.216 (−1.939, −0.494)	0.001	0.736	0.001
Frequent use of salt^a^	0.109 (−1.444, 1.663)	0.890	−2.843 (−3.901, −1.784)	<0.001	−1.049 (−2.540, 0.442)	0.168	0.487	0.404
Frequent intake of animal internal organs^b^	0.047 (−1.240, 1.335)	0.942	−0.080 (−0.635, 0.475)	0.778	−1.469 (−2.351, −0.587)	0.001	0.904	0.768

^a^At least once per day

^b^More than twice per month

^c^Adjusted for age, sex, education level, relationship status, occupation, smoking status and alcohol consumption

^d^Commune random effect

^e^Participant random effect

## Discussion

In this study, Vietnamese adults with metabolic syndrome were identified from individuals initially screened and recruited from the community. The final sample of 337 participants at the post-intervention evaluation represented an overall retention rate of 80.8%, which was higher than in previous studies [22, 35]. The low attrition may reflect the acceptability of the Vietnam Physical Activity and Nutrition programme to the participants. Indeed, the group leaders were specifically trained to improve retention and engagement of participants in their walking groups, while the physical activity and healthy eating information provided in the booklet and education sessions was relevant and appropriate for the target group. Such strategies have been found to boost retention successfully in intervention studies [36, 37].

The results demonstrated changes in physical activity and dietary behaviours among the intervention participants when compared with the controls. Our findings were consistent with those from previous studies in terms of physical activity and nutrition outcomes [18, 22, 35, 38]. For example, a recent home-based intervention on Australian adults with, or at risk of, metabolic syndrome reported a significant increase in moderate activity and a reduction in sitting time among intervention participants [22]. In particular, the Vietnam Physical Activity and Nutrition programme had led to significant improvements in moderate activity participation, walking time and total physical activity, as well as a reduction in sitting time for the intervention group. In addition, data recorded by pedometers confirmed a substantial increase of 5160 steps taken on average after the intervention, consistent with findings from a systematic review and meta-analysis [39]. Significant improvements in waist circumference (−1.63 cm, *P* < 0.001) and weight (−1.44 kg, *P* < 0.001) among the intervention group compared with the control group after controlling for the effects of clustering and confounding factors were also found [40].

The Vietnam Physical Activity and Nutrition programme followed the WHO’s Recommendations for Physical Activity [41], encouraging participants to undertake at least 150 min of moderate intensity activity per week, or equivalent. This message was reinforced during the education sessions, while individuals were guided to tailor the programme to suit their own needs, such as walking more or walking less. Advice and regular feedback were provided by the walk leaders and programme facilitators to monitor dietary and physical activity behaviours [14, 37]. The adopted approach not only supported participants but also enabled them to manage their own progress, thereby increasing their sense of ownership of the Vietnam Physical Activity and Nutrition programme. Walking in groups has been shown to increase moderate physical activity among adults. It is accessible for everyone and is suitable for all socio-economic groups [15] especially older adults [16, 17], even those with chronic diseases [15]. The dramatic increase in walking among the intervention participants suggested the suitability of the walking group for Vietnamese adults with metabolic syndrome.

The nutrition component of the Vietnam Physical Activity and Nutrition programme was developed based on the Food-Based Dietary Guidelines in Vietnam [42], which encouraged participants to eat more vegetables and fruits every day, reduce the amount of salt and cooking oil used when preparing meals, and reduce the consumption of animal internal organs. It also advised participants to eat boiled meals instead of stir-fried or deep-fried foods, together with tips on how to adhere to these guidelines, and goal setting. The intervention resulted in slight increases in the intake of daily fruit and vegetables, but since most participants already reported consumption at least once per day at baseline, further improvement was somewhat limited by the ‘ceiling effect’ [38]. However, significant reductions were achieved in the use of cooking oil (*P* = 0.001) and the consumption of animal internal organs (*P* = 0.001).

Understanding the barriers and enablers that influence physical activity and dietary behaviours can assist in the development of appropriate health promotion interventions [43]. The Vietnam Physical Activity and Nutrition programme undertook formative research to identify and address barriers that were subsequently incorporated into the programme. Experience, lessons and suggestions from other participants, as well as facilitators, on overcoming the barriers and on insights into enablers, were discussed throughout the education sessions and implemented in the programme.

Creating a supportive environment and establishing a network of new friends through the walking groups and educations sessions also enhanced positive behaviour changes. These strategies have previously been documented to improve physical activity [44] and might contribute to the improved outcomes for this study. Although the Hawthorne effect might affect behavioural changes [45], such an impact was expected to be minor for randomized controlled trials [46, 47].

### Limitations

There are several limitations in this study. The intervention programme was followed up for 6 months, in line with recommendation for metabolic syndrome control under supervision [48]. Assessment of sustainability of the programme and behavioural changes over a longer term is not feasible owing to budget constraint and resource limitations. Although demographic and other factors were controlled for in the mixed regression analyses, residual confounding may still exist and potentially affect the results. Another shortcoming concerned the objective measurement of physical activity, whereby pedometers were provided to the intervention participants only to motivate walking. The use of objective physical activity measures,such as pedometers and accelerometers, in both intervention and control groups should be considered in future research.

## Conclusions

The Vietnam Physical Activity and Nutrition programme was the first physical activity and nutrition intervention specifically targeting Vietnamese adults with metabolic syndrome. This cluster-randomized controlled trial demonstrated increases in moderate intensity activity, walking and total physical activity, as well as reductions in sitting time, intake of animal internal organs and using cooking oil for daily meal preparation among the intervention participants, when compared with the control group over a 6 month period. The findings confirmed that the prescribed community-based intervention with supportive environments can effectively improve physical activity and dietary behaviours for adults with metabolic syndrome in Vietnam.

## Additional file

Additional file 1: CONSORT checklist of the trial. (PDF 138 kb)

## Abbreviations

CONSORT: Consolidated Standards of Reporting Trials
MET: metabolic equivalent task
WHO: World Health Organization
